# 

**Published:** 1990-06

**Authors:** 


					Wl WE403
I'nfL- N?'2 1S90
1 SEQ: SR00 68788
"l* WEST OF ENGLAND MEBICAI
JOURNAL
Volume 105(ii)
June 1990
Cerebellar Glioblastoma
Intra-ocular Lenses
Pulmonary uptake of MDP
Intra-operative Ultrasound in Surgery of Isulinomas
Some Plymouth Wprthies (Part 2)
The Medicine of the People
A West Country Composer
Dartmoor: A Place to Explore
TOEMimiLY IBEIOTdDL MIEBE(D?=C1HIIIETUK<K1IC!AIL JOTOMAIL
ISSN: 0960 6440 NLM Code B5Q

				

